# Syncope as a sign of occult cancers: a population-based cohort study

**DOI:** 10.1038/s41416-019-0692-2

**Published:** 2019-12-20

**Authors:** Mads Okkels Birk Lorenzen, Dóra Körmendiné Farkas, Kasper Adelborg, Jens Sundbøll, Henrik Toft Sørensen

**Affiliations:** 1 Department of Clinical Epidemiology, Aarhus University Hospital, Aarhus, Denmark; 2 Department of Clinical Biochemistry, Aarhus University Hospital, Aarhus, Denmark

**Keywords:** Cancer epidemiology, Risk factors

## Abstract

**Background:**

We examined if syncope was a marker of an occult cancer by comparing the risk in patients with a syncope episode with that of the general population.

**Methods:**

Using Danish population-based medical registries, we identified all patients diagnosed with syncope during 1994–2013 and followed them until a cancer diagnosis, emigration, death or end of follow-up, whichever came first. We computed cumulative risks and standardised incidence ratios (SIR) with 95% confidence intervals (CI).

**Results:**

Among 208,361 patients with syncope, 20,278 subsequent cancers were observed. The 6-month cumulative risk of any cancer was 1.2%, increasing to 17.9 % for 1–20 years of follow-up. The highest cumulative risks after 6 months of follow-up were lung cancer (0.2%), colorectal cancer (0.2%), prostate cancer (0.1%) and brain cancer (0.1%). The 6-month SIR were 2.7 (95% CI: 2.4–3.0) for lung cancer, 2.0 (95% CI: 1.8–2.2) for colorectal cancer, 1.7 (95% CI: 1.5–1.9) for prostate cancer and 10.0 (95% CI: 8.6–11.4) for brain cancer.

**Conclusions:**

Syncope was a weak marker of an occult cancer. In short-term the highest cumulative risks were observed for lung, colorectal, prostate and brain cancers. An aggressive search for occult cancer in a patient with syncope is probably not warranted.

## Background

Syncope is defined as a sudden loss of consciousness of short duration, with an inability to maintain postural tone, and spontaneous complete recovery.^1,2^ Episodes occur frequently, accounting for ~1% of all referrals to emergency departments.^1^ The lifetime cumulative risk of syncope is approximately 35%.^3^ Clinically, the condition is categorised on the basis of underlying pathophysiology, i.e. reflex-mediated, orthostatic hypotension or cardiac syncope.^1^ Whether syncope may be the presenting sign of an occult cancer is largely unknown. Only a few case reports are available.^4–17^ Thus, firm epidemiological evidence on the association between syncope and cancer is lacking.

Syncope can occur due to stimulation of the parasympathetic nervous system or carotid sinus by direct neoplastic infiltration.^7,9–16^ Syncope also can be the first sign of intracranial tumours due to involvement of autonomic cardiovascular control areas.^4,18–20^ Additionally, a recent multicentre study showed that pulmonary embolism was identified in nearly one of every six patients hospitalised for a first episode of syncope,^21^ and pulmonary embolism, in turn, is established as a marker for occult cancer.^22,23^ Therefore, it is possible that syncope may be associated with an underlying undiagnosed cancer. Electrolyte imbalance and paraneoplastic phenomena also may induce syncope, as observed in patients with pheochromocytoma,^24^ mastocytosis,^25,26^ and carcinoid syndrome.^27^

To study these issues in detail, we examined overall risk of cancer and risk of site-specific cancers in a large cohort of syncope patients, and we compared their cancer risk with that of the general population.

## Methods

### Design and setting

We conducted a nationwide population-based cohort study in Denmark between 1 January 1994 and 30 November 2013. The Danish national health system provides tax-supported healthcare to all residents of Denmark, ensuring equal access to general practice and hospital care.^28^ Contacts with the healthcare system are recorded in national databases.^29,30^ Linkage among databases is possible through a unique ten-digit personal identification number assigned to each Danish resident.^29^

### Patients with syncope

We used the Danish National Patient Registry (DNPR) to identify all patients with a first-time inpatient, outpatient or emergency room diagnosis of syncope. We excluded patients with a history of a cancer diagnosis recorded in the Danish Cancer Registry (DCR). The DNPR contains data on all inpatient admissions to non-psychiatric hospitals since 1977 and on hospital outpatient and emergency room contacts since 1995. Information recorded in the DNPR includes admission and discharge dates, and one primary and up to nineteen secondary discharge diagnoses coded according to the International Classification of Diseases (ICD) Tenth Revision since 1994.^30^ The syncope diagnosis has previously been reported with a positive predictive value of 95%, and thus diagnostic misclassification of the exposure is likely negligible.^31^

### Cancer

To obtain information on incident cancers diagnosed after a syncope episode, we linked our patient cohort to the DCR.^32^ This registry contains detailed information on cancers diagnosed in Denmark since 1943, with information on morphology, histology and cancer stage at diagnosis.^33^ We categorised the cancers according to recommendations from the Danish National Board of Health^34^ (Supplementary Table 1).

### Comorbidity

Information on several comorbidities was obtained from the DNPR. These included diagnoses of head trauma, diabetes mellitus, myocardial infarction, heart failure, atrial fibrillation, valvular heart disease, chronic lower respiratory diseases, chronic kidney disease, obesity, alcoholism-related disorders, epilepsy, narcolepsy and cataplexy, stroke, angina pectoris, hypertension, anaemia, lower urinary tract obstruction and venous thromboembolism. Changes in vital and migration status were ascertained from the Danish Civil Registration System, which is electronically updated on a daily basis.^29^ All ICD codes used in the study are provided in Supplementary Table 1.

### Statistical analyses

We followed the patients from their syncope episode (hospital contact date) until a cancer diagnosis, death, emigration, or 30 November 2013, whichever came first. We tabulated characteristics of the syncope cohort and calculated cumulative risks (%) of a cancer diagnosis during 0–6 months, >6–12 months and 1–20 years following a syncope episode, treating death as a competing risk.^35,36^ Assuming that the observed number of cancers followed a Poisson distribution, we calculated relative risks by comparing the observed number to the expected number of cancers (based on national incidence rates, by sex, age and calendar year, in 1 year intervals) to obtain a standardised incidence rate (SIR) with 95% confidence intervals (CIs). The SIR analysis also takes into account the competing risk of death, as individuals are censored when they die and because the national cancer incidence rates are based exclusively on individuals at risk of cancer, censoring individuals who die or emigrate. Exact 95% CIs were used when the observed number of cancers was less than ten.^35,37^ We stratified the main analyses (SIRs) by follow-up time (0–6 months, >6–12 months, >12 months and 0–20 years), age (0–29 years, 30–49 years, 50–69 years and >70 years), calendar period (1994–1998, 1999–2003, 2004–2008 and 2009–2013) and sex. In addition, we stratified the analyses by presence or absence of the individual comorbidities presented in Table 1 (except for narcolepsy and cataplexy, due to their low prevalence). This analysis was performed only for the four most common cancers to retain statistical precision of the estimates (Supplementary Table 2–5). We calculated the number of patients needed to be screened to find 1 excess cancer as the reciprocal of excess risk, assuming that the cancer diagnosed within 6 months after the syncope diagnosis was already present at the syncope diagnosis.

**Table 1:** Characteristics of the syncope cohort, Denmark, 1994–2013.

*Total number*	208,361 (100)
Sex	
Male	97,135 (47)
Female	111,226 (53)
Type of contact	
Inpatients	104,420 (50)
Outpatients	27,427 (13)
Emergency patients	76,514 (37)
Type of diagnosis	
Primary diagnosis^a^	182,869 (88)
Secondary diagnosis	25,492 (12)
*Median age, years (25th–75th percentiles)*	57 (32–74)
Age group, years	
0–29 years	48,143 (23)
30–49 years	38,257 (18)
50–69 years	56,731 (27)
≥70 years	65,230 (31)
Calendar period	
1994–1998	39,622 (19)
1999–2003	51,815 (25)
2004–2008	56,501 (27)
2009–2013	60,423 (29)
*Median follow-up time, years (25th–75th percentiles)*	56 (2–10)
Comorbidities	
Head trauma	58,958 (28)
Diabetes mellitus	11,484 (6)
Myocardial infarction	12,869 (6)
Heart failure	11,400 (6)
Atrial fibrillation	17,637 (8)
Valvular heart disease	4903 (2)
Chronic lower respiratory diseases	15,805(8)
Chronic kidney disease	3961 (2)
Obesity	6457 (3)
Alcoholism-related disorders	10,309 (5)
Epilepsy	10,093 (5)
Narcolepsy and cataplexy	48 (0)
Stroke	14,829 (7)
Angina pectoris	18,287 (9)
Hypertension	26,771 (13)
Anemia	2171 (1)
Lower urinary tract obstruction	5387 (3)
Venous thromboembolism	4986 (2)

Patients also can be assigned one or more appropriate secondary diagnoses Data are numbers (%)

^a^Primary diagnosis refer to the primary reason for a hospital contact

All statistical analyses were conducted using SAS, version 9.4 (SAS Institute, Cary, NC). The study was approved by the Danish Data Protection Agency (record number: 1-16-02-1-08). In Denmark, registry-based research does not require permission from an ethics committee or informed consent from patients.

## Results

We identified 208,361 patients with a first-time episode of syncope. Among these patients, 47% were male, 88% had syncope as their primary reason for the index hospital contact. In the syncope cohort, 50% were inpatients, 13% outpatients and 37% emergency room contacts. The median age was 57 years and median follow-up time was 5.6 years. Head trauma, cardiovascular disease (hypertension, atrial fibrillation and angina pectoris) and chronic lower respiratory diseases were the most frequent comorbidities (Table 1).

We observed 20,278 cancers during 20 years of follow-up. The cumulative risk of any cancer diagnosis after 6 months of follow-up was 1.2%, increasing to 17.9% for 1–20 years of follow-up (Table 2). The 6-month cumulative risk of cancer was mainly driven by lung cancer (0.2%), colorectal cancer (0.2%), prostate cancer (0.1%) and brain cancer (0.1%). The 6-month cumulative risks were low for haematological malignancies (below 0.1% for all groups of cancers) (Table 2)

**Table 2:** Cumulative risks of cancer among patients with syncope by follow-up time, treating death as competing risk.

Type of cancer	0–6 months	>6–12 months	1–20 years
*All*	1.2 (1.2–1.3)	0.7 (0.7-0.7)	17.9 (17.3–18.3)
Intracranial tumours			
Meninges	0.1 (0.0–0.1)	<0.1^d^	0.2 (0.1–0.2)
Brain	0.1 (0.1–0.1)	<0.1^d^	0.4 (0.3–0.5)
Supradiaphragmatic tumours			
Pharynx^a^	<0.1^d^	<0.1^d^	0.2 (0.2–0.3)
Lung	0.2 (0.2–0.2)	0.1 (0.1–0.1)	1.8 (1.7–2.0)
Tongue	<0.1^d^	<0.1^d^	0.1 (0.0–0.1)
Oral cavity	<0.1^d^	<0.1^d^	0.1 (0.1–0.2)
Breast	0.1 (0.1–0.1)	0.1 (0.1–0.1)	1.5 (1.4–1.7)
Infradiaphragmatic tumours			
Liver	<0.1^d^	<0.1^d^	0.3 (0.2–0.4)
Stomach	<0.1^d^	<0.1^d^	0.2 (0.2–0.3)
Kidney	<0.1^d^	<0.1^d^	0.2 (0.2–0.3)
Colorectal^b^	0.2 (0.1–0.8)	0.1 (0.1–0.1)	1.9 (1.7–2.0)
Oesophagus	<0.1^d^	<0.1^d^	0.2 (0.2–0.3)
Prostate	0.1 (0.1–0.1)	0.1 (0.1–0.1)	1.8 (1.7–2.0)
Pancreas	<0.1^d^	<0.1^d^	0.4 (0.3–0.4)
Ovary	<0.1^d^	<0.1^d^	0.2 (0.1–0.2)
Urinary bladder	0.1 (0.0–0.1)	<0.1^d^	0.9 (0.8–1.0)
Haematology malignancies			
Non-Hodgkin lymphoma incl. multiple myeloma	0.1 (0.0–0.1)	<0.1^d^	0.6 (0.5–0.7)
Leukaemia^c^	<0.1^d^	<0.1^d^	0.3 (0.2–0.3)

Data are percentages with 95% confidence intervals

^a^Pharyngeal cancers include cancers in the nasal part of the pharynx, pharyngeal tonsil, the pharyngeal cavity, and other parts of the pharynx (unspecified, Waldeyer’s ring, and overlapping lesions of the lip, oral cavity, and pharynx)

^b^Colorectal cancer includes cancers of the rectum, caecum, anus, appendix, ascending colon hepatic flexure, transverse colon, splenic flexure, descending colon, sigmoid colon, and colon unspecified

^c^Leukaemia includes: monocytic leukaemia, acute myeloid leukaemia (acute myeloid leukaemia with multilineage dysplasia and myeloid leukaemia, unspecified), and other leukaemias of specified cell type. Lymphoid leukaemia: acute leukaemias, chronic lymphocytic leukaemia of B-cell type, prolymphocytic leukaemia of B-cell type, hairy-cell leukaemia, adult T-cell lymphoma/leukaemia, prolymphocytic leukaemia of T-cell type, other lymphoid leukaemia, mature B-cell leukaemia Burkitt-type, and lymphoid leukaemia, unspecified

^d^Cumulative risk below 0.1% due to low number of outcomes

.

The 6-month SIR for any cancer was 2.0 (95% CI: 1.8–2.0), while the >6–12 month and >12-month SIRs were 1.1 (95% CI: 1.0–1.1) and 1.0 (95% CI: 1.0–1.0), respectively (Table 3). The overall cancer SIR was slightly higher during 2009–2013 than during 1994–1998. It was also higher among patients aged 30–49 years [SIR = 1.2 (95% CI: 1.1–1.2)] than among other age groups. In addition, the SIR was slightly higher among men [SIR = 1.1 (95% CI: 1.1–1.2)] than among women [SIR = 1.1 (95% CI: 1.0–1.1)] (Table 3). The 6-month SIRs were 2.7 (95% CI: 2.4–3.0) for lung cancer, 2.0 (95% CI: 1.8–2.2) for colorectal cancer, 1.7 (95% CI: 1.5–1.9) for prostate cancer, and 10.0 (95% CI: 8.6–11.4) for brain cancer (Table 4).

**Table 3:** Observed vs. expected cancers in the syncope cohort and standardised incidence ratios (SIRs) in analyses stratified by age, calendar period, and sex.

	Observed/expected	SIR (95% confidence interval)
Any cancer		
0–6 months	2541/1333	2.0 (1.8–2.0)
>6–12 months	1328/1245	1.1 (1.0–1.1)
>12 months	16,409/16,029	1.0 (1.0–1.0)
0–20 years	20,278/18,607	1.1 (1.1–1.1)
Age groups, years		
0–29	384/330	1.2 (1.1–1.3)
30–49	1844/1573	1.2 (1.1–1.2)
50–69	8356/7570	1.1 (1.1–1.1)
70+	9694/9134	1.1 (1.0–1.1)
Calendar period		
1994–1998	5936/5605	1.1 (1.0–1.1)
1999–2003	6686/6253	1.1 (1.0–1.1)
2004–2008	5315/4879	1.1 (1.1–1.1)
2009–2013	2341/1870	1.3 (1.2–1.3)
Sex		
Female	9157/8727	1.1 (1.0-1.1)
Male	11,121/9880	1.1 (1.1-1.2)

**Table 4:** Risk of site-specific cancers after syncope, by follow-up period.^a^

Site	0–6 months		>6–12 months		>12 months		0–20 years	
	Observed/expected	SIR (95% CI)	Observed/expected	SIR (95 % CI)	Observed/expected	SIR (95% CI)	Observed/expected	SIR (95% CI)
Intracranial tumours								
Meninges	93/8	11.4 (9.2–13.4)	19/8	2.5 (1.5–3.9)	157/106	1.5 (1.3–1.8)	269/121	2.2 (2.0–2.5)
Brain	231/23	9.99 (8.6–11.4)	46/22	2.1 (1.6–2.8)	338/284	1.2 (1.2–1.3)	615/329	1.9 (1.7–2.0)
Supradiaphragmatic tumours								
Pharynx^b^	17/7	2.3 (1.4–3.7)	12/7	1.8 (0.9–3.1)	182/96	1.9 (1.6–2.2)	211/110	1.9 (1.7–2.2)
Lung	366/135	2.7 (2.4–3.0)	134/125	1.1 (0.9–1.3)	1655/1541	1.1 (1.0–1.1)	2155/1802	1.2 (1.2–1.3)
Tongue	9/3	2.6 (1.2–4.9)	7/3	2.2 (0.9–4.4)	56/44	1.3 (1.0–1.7)	69/48	1.4 (1.1–1.8)
Oral cavity	13/6	2.1 (1.1–3.7)	13/6	2.3 (1.2–3.9)	116/71	1.6 (1.3–2.0)	142/83	1.7 (1.4–2.0)
Breast	140/114	1.2 (1.0–1.5)	110/108	1.0 (0.8–1.2)	1407/1424	1.0 (0.9–1.0)	1657/1646	1.0 (1.0–1.1)
Infradiaphragmatic tumours								
Liver	41/11	3.8 (2.8–5.2)	16/10	1.6 (0.9–2.6)	162/124	1.3 (1.1–1.5)	219/145	1.5 (1.3–1.7)
Stomach	60/20	2.9 (2.6–3.8)	29/19	1.5 (1.0–2.2)	212/220	1.0 (0.8–1.1)	301/259	1.2 (1.0–1.3)
Kidney	49/19	2.6 (1.9–3.5)	24/17	1.4 (0.9–2.1)	229/219	1.0 (0.9–1.2)	302/256	1.2 (1.1–1.3)
Colorectal^c^	308/155	2.0 (1.8–2.2)	149/145	1.0 (0.9–1.2)	1812/1765	1.0 (1.0–1.1)	2269/2065	1.1 (1.1–1.6)
Oesophagus	27/15	1.8 (1.2–2.7)	18/14	1.3 (0.8–2.1)	220/169	1.3 (1.1–1.5)	265/198	1.3 (1.2–1.5)
Prostate	228/135	1.7 (1.5–1.9)	120/126	1.0 (0.8–1.1)	1610/1649	1.0 (0.9–1.0)	1958/1910	1.0 (1.0–1.1)
Pancreas	48/31	1.6 (1.1–2.1)	17/29	0.6 (0.3–0.9)	375/361	1.0 (0.9–1.2)	440/421	1.0 (1.0–1.2)
Urinary bladder	93/69	1.4 (1.1–1.7)	79/64	1.24 (1.0–1.6)	787/748	1.1 (1.0–1.1)	959/879	1.1 (1.0–1.2)
Haematology malignancies								
Non-Hodgkin lymphoma incl. multiple myeloma	100/42	2.4 (2.0–2.9)	40/39	1.0 (0.7–1.4)	516/495	1.0 (1.0–1.1)	656/576	1.14 (1.05–1.23)
Leukaemia^d^	54/28	1.9 (1.5–2.5)	30/26	1.2 (0.8–1.7)	275/317	0.9 (0.8–1.0)	359/371	1.0 (0.9–1.1)
Cancers with no associations								
Ovary	22/16	1.3 (0.8–2.0)	14/15	0.9 (0.5–1.5)	171/187	0.9 (0.8–1.1)	207/218	1.0 (0.8–1.1)
Melanoma	41/33	1.3 (0.90–1.7)	37/31	1.2 (0.8–1.7)	438/447	1.0 (0.9–1.1)	516/510	1.0 (0.9–1.1)
Basal cell carcinoma of the skin	258/262	1.0 (0.9–1.1)	228/245	0.9 (0.8–1.1)	3271/3412	1.0 (0.9–1.0)	3757/3921	1.0 (0.9–1.0)

Data are standardised incidence ratios (SIRs) with 95% confidence intervals (CI)

^a^Only cancer sites with total number of outcomes above 50 is presented in this table

^b^Pharynx includes cancers in the nasal part of the pharynx, pharyngeal tonsil, the pharyngeal cavity, and other parts of pharynx (unspecified, Waldeyer’s ring, and overlapping lesions of the lip, oral cavity, and pharynx)

^c^Colorectal cancer includes cancers in the rectum, caecum, anus, appendix, ascending colon hepatic flexure, transverse colon, splenic flexure, descending colon, sigmoid colon, and colon unspecified

^d^Leukaemia includes: monocytic leukaemia, acute myeloid leukaemia (acute myeloid leukaemia with multilineage dysplasia and myeloid leukaemia, unspecified), and other leukaemias of specified cell type. Lymphoid leukaemia: acute leukaemias, chronic lymphocytic leukaemia of B-cell type, prolymphocytic leukaemia of B-cell type, hairy-cell leukaemia, adult T-cell lymphoma/leukaemia, prolymphocytic leukaemia of T-cell type, other lymphoid leukaemia, mature B-cell leukaemia Burkitt-type, and lymphoid leukaemia, unspecified

In stratified analyses we found that the SIR of cancer (except breast cancer) was higher for those with anaemia than those without anaemia (Supplementary Table 2–5). For example, the 6-month SIR of colorectal cancer was 3.4 (95% CI: 1.5–6.7) for patients with anaemia, while it was 2.0 (95% CI: 1.8–2.2) for those without anaemia (Supplementary Table 3). For syncope patients with and without lower urinary tract obstruction, the cancer SIRs were fairly comparable, although the 6-month SIR of prostate cancer was 3.3 (95% CI: 2.3–4.6) for patients with lower urinary tract obstruction and 1.5 (95% CI: 1.3–1.8) for those without (Supplementary Table 5). The cancer SIRs were broadly similar for patients with and without venous thromboembolism (Supplementary Table 2–5).

During 6 months of follow-up, we found 1208 excess cancers (2541 observed–1333 expected) (Table 3). The number of patients needed to be screened among the syncope cohort to find 1 excess cancer was 172 (208,361 total/1208 excess cancers).

## Discussion

In this large population-based cohort study, syncope was a weak marker of an occult cancer. The cumulative risks were low and after 6 months of follow-up, it was mostly elevated for lung, colorectal, prostate and brain cancers.

The association between syncope as marker of occult cancer has been sparsely documented in the literature. Several case reports found that syncope was the presenting symptom of lung cancer^10–12^ and brain cancer.^3–5^ In contrast, such reports were not found for colorectal or prostate cancers. This may indicate that cancer-induced syncope is more frequently due to undiagnosed lung cancer than colorectal or prostate cancer. Our findings support this notion, i.e., the most frequent occult cancers found in the syncope cohort were cancers of the lung, particularly in the short-term. Case reports describing syncope as the initial symptom of a brain tumour also are in line with our findings.^3–5^ Although several case reports described syncope as a symptom of head and neck cancer^7,13–16^ or an undiagnosed haematological malignancy,^38–41^ our findings suggest that the cumulative risks were low for these conditions.

Cancer might be associated with syncope for several pathophysiological reasons. Lung cancer may cause syncope due to cerebral hypoperfusion or brain metastases. Cerebral hypoperfusion may be induced by compression of blood vessels by tumours, reducing blood flow to the heart or brain, or by direct infiltration of the vagus and glossopharyngeal nerves.^10^ Although the mechanisms are not well understood, brain metastases from lung cancer may involve areas responsible for cardiovascular control (brainstem, thalamus, hypothalamus, insular cortex or amygdala) and thus cause syncope.^4,42,43^ This is supported by case reports of frontal and temporal lobe tumours, as well as craniocervical junction tumours, which provide the best explanation for bradycardia and/or cardiac asystole and subsequent syncope.^18–20^ It is also possible that invasion or compression of vessels by a tumour reduce blood flow to the heart and brain, inducing cerebral hypotension. However, only one case report on infradiaphragmatic tumours in the form of renal cell carcinoma has supported this explanation.^8^ Another possibility is metastases from a colorectal cancer to the lung and brain; however, this is not a frequent complication.^44,45^ Furthermore, anaemia may play a role in inducing syncope,^46^ and is often a complication to cancer.^47^ This notion was supported by our stratified analyses, although the prevalence of anaemia likely is underestimated in our population. This misclassification implies that the impact of anaemia might be even higher than observed in our data. Another well-known syncope-inducing mechanism is micturition.^48^ It is thought that patients with lower urinary tract obstruction might experience micturition syncope.^49^ Venous thromboembolism is also a well-known complication to cancer, and can also be a cause of syncope.^50,51^ However, in our data the cancer SIRs were fairly similar for those with and without venous thromboembolism, suggesting that venous thromboembolism may play a minor role only in the association between syncope and cancer.

As documented in our analyses, syncope may be the first sign of some cancers. Occult cancer diagnosed at the short-term follow-up following syncope likely represent aggressive and fast-growing tumours (i.e. lung cancer and colorectal), or slow-growing tumours that have gradually become large, until a certain point, where they will facilitate a syncope episode. As discussed previously it could involve e.g. the lower urinary system, compression of blood vessels to the heart and brain, or a slowly evolving anaemia.

At long-term follow-up, the predominant cancers are most likely slow-growing tumours (e.g. carcinoid tumours) that have been present for years without noticeable symptoms. At one point, they may hemodynamically destabilise the patient and induce a syncope.

The main strength of our study is its nationwide population-based cohort design within a setting of free and equal access to healthcare service, which limits selection and referral biases.^29^ As well, coverage of cancer diagnoses is highly complete in the Danish Cancer Registry.^33^ Our study also has several potential limitations. Heightened diagnostic effort may explain in part the short-term cancer risk. However, if diagnostic bias was prominent, we would expect a drop in overall SIR estimates (to below 1) after 6 months as a compensatory deficit. As this pattern not was observed in our analyses, heightened diagnostic efforts are unlikely to explain our findings. We lacked clinical detail and the exact benefit of searching for cancer in a patient with a syncope is therefore difficult to assess. In our cohort, most of the cancers that were found during the first year of follow-up were probably present at the time of the syncope. The detection of some of these cancers would have required an extensive diagnostic workup, and it is unclear whether early diagnosis would have changed the outcome. For several of the types of cancer, such as brain cancer, early detection may not necessarily change the prognosis. Other cancers might be detected by simple methods. In the group we studied, 208,361 persons would have had to be screened for the 1208 excess cancers to be found during the first 6 months of follow-up. It should be noted that we in this calculation assumed a screening sensitivity of 100%, which is rarely the case. In addition, the screening sensitivity may differ among different cancers types. It is also unknown if formal screening for cancer symptoms among syncope patients will benefit their prognosis. Therefore, extensive cancer screening of patients with syncope does not seem to be cost effective.

## Conclusion

In this population-based study, an episode of syncope was a weak marker of an occult cancer diagnosed within the following 6 months, mainly driven by lung, colorectal, prostate and brain cancers.

## Supplementary information

Supplemental material

## Data Availability

Danish law does not allow researchers to share raw data or datasets which include individual-level datapoints from the registries with third parties. Data can be accessed by researchers through application to the Health Data Authority (contact:forskerservice@sundhedsdata.dk). However, a formal affiliation or collaboration with a Danish research institution is required. Acquisition of data are only allowed after permission to handle data has been obtained from the Danish Data Protection Agency (contact: dt@datatilsynet.dk).
