# Supplementary material for: Syncope as a sign of occult cancers: a population-based cohort study
Source: Br J Cancer. 2019 Dec 20;122(4):595–600. doi: 10.1038/s41416-019-0692-2 (PMC7029007; doi:10.1038/s41416-019-0692-2)
Supplement: Supplementary file 1 — Supplemental material [file 41416_2019_692_MOESM1_ESM.docx]

| **Supplementary Table 1. *International Classification of Diseases, Eighth Revision* (ICD-8) and *Tenth Revision* (ICD-8 & 10) codes used in the study.** | | |
| --- | --- | --- |
| Diagnosis | ICD-8 | ICD-10 |
| Syncope and collapse | 782.5 | R55 |
| Cancers: |  |  |
| Lip |  | C00 |
| Tongue |  | C01–02 |
| Oral cavity |  | C03–06 |
| Salivary gland |  | C07–C08 |
| Tonsil and pharynx |  | C09–C13 |
| Other and poorly specified locations in the lip, oral cavity, or pharynx |  | C14 |
| Esophagus |  | C15 |
| Stomach |  | C16 |
| Small intestine |  | C17 |
| Colorectal |  | C18-C19 |
| Anus |  | C20-C21 |
| Liver including intrahepatic bile ducts |  | C22 |
| Gallbladder and bile ducts |  | C23–C24 |
| Pancreas |  | C25 |
| Other and ill defined cancers of digestive organs |  | C26 |
| Nasal cavity, middle ear, and accessory sinuses |  | C30–C31 |
| Larynx |  | C32 |
| Lung, bronchus and trachea |  | C33–C34 |
| Thymus |  | C37 |
| Heart and mediastinum |  | C380–383, C388 |
| Pleura including mesothelioma pleura |  | C384, C450 |
| Other and poorly specified locations in airways and respiratory organs |  | C39 |
| Bone and articular cartilage |  | C40–C41 |
| Melanoma |  | C43 |
| Non-melanoma skin cancer |  | C44 |
| Mesothelioma |  | C45.1–C45.9 |
| Kaposi’s sarcoma |  | C46, B210 |
| Peripheral nerves and autonomic nervous system |  | C47 |
| Retroperitoneum and peritoneum, and malignant neoplasm |  | C48 |
| Other connective and soft tissue |  | C49 |
| Breast |  | C50 |
| External female genitalia excluding basal cell carcinomas (morphological code 809) |  | C51 |
| Vagina excluding basal cell carcinomas (morphological code 809) |  | C52 |
| Cervix |  | C53 |
| Uterus |  | C54–C55 |
| Ovary and fallopian tube |  | C56, C570–574 |
| Placenta |  | C58 |
| Other and unspecified female genital organs |  | C577–579 |
| Penis excluding basal cell carcinomas (morphological code 809) |  | C60 |
| Prostate |  | C61 |
| Testicle |  | C62 |
| Other and unspecified cancers in male genital organs excluding basal cell carcinomas (morphological code 809) |  | C63 |
| Kidney |  | C64 |
| Renal pelvis |  | C65, D301, D411 |
| Ureter |  | C66, D302, D412 |
| Urinary bladder |  | C67, D090, D303, D414 |
| Other and unspecified urinary organs |  | C68, D091, D304–309, D413, D417–419 |
| Eye |  | C69 |
| Meninges |  | C70 ,D32,D42 |
| Brain including hypophysis, corpus pineale, and ductus craniopharyngealis |  | C71, C751–C753, D330–D332, D352–D354, D430–D432, D443–D445 |
| Spinal cord, cranial nerves, and other parts of central nervous system |  | C72 D333–D339, D433–D439 |
| Thyroid |  | C73 |
| Adrenal gland |  | C74 |
| Malignant neoplasm of other endocrine glands and related structures |  | C750, C754-759 |
| Hodgkin’s lymphoma |  | C81 |
| Non-Hodgkin’s lymphoma incl. Multiple myeloma |  | C82–C86, C90 |
| Malignant myeloproliferative disease |  | D47.1, D47.3, D47.4, D47.5, D45 |
| Leukaemia |  | C91,C92, C93,C94-C96 |
| Metastasis and unspecified cancer in lymph nodes (only if no primary tumor is coded) |  | C77–C79 |
| Malignant neoplasm of other ill- defined or unspecified sites |  | C76, C80 |
| Comorbidity | | |
| Head trauma | 800-803, 850-854, 810-874 | S00-S09 |
| Diabetes mellitus | 249.00, 249.06, 249.07, 249.09, 250.00, 250.06, 250.07, 250.09 | E10-E14 (except E102, E112, E142). O24 (except O24.4), G63.2, H36.0, N08.3. |
| Myocardial infarction | 410 | I21, I22, I23 |
| Heart failure | 427.09; 427.10; 427.11; 427.19; 428.99; 782.49 | I50; I11.0; I13.0; I13.2 I420, I426, I427, I428, I429 |
| Atrial fibrillation | 427.93, 427.94 | I48 I47,I49 |
| Chronic lower respiratory diseases | 490–493, 515–518 | J40–J47, J60–J67,  J684, J701, J703, J841,  J920, J961, J982, J983 |
| Valvular heart diseases | 394 - 395 | I05, I34, I390, I511A, I06, I35, I391 |
| Chronic kidney disease | 24902, 25002, 75310-75319, 582-584, 59009, 59320, 792 | N00, N01, N03, N05, I12, I13, I15.0, I15.1, N11, N14, N15, N16, Q61.1-Q61.4, E10.2, E11.2, E14.2, N08.3, N18-N19, N26, N27, N07, N08 |
| Obesity | 277 | E65-E68 |
| Epilepsy | 345.31, 345.30, 345.38, 345.39, 345.00, 345.09, 345.10, 345.11, 345.18, 345.19, 345.20, 345.29, 345.32, 345.33, 345.9 | G40, G41, G43, G44, |
| Narcolepsy and cataplexy | 347.0 | G47.4 |
| Stroke, Subarachnoid haemorrhage, Intracerebral haemorrhage, Transient cerebral ischaemic attacks | 43000-43099, 43100, 43108–43190, 43198-43199, 43200–43299, 43309–43399, 43409-43499, 43600-43699, 43509-43599 | G45, G47.4, I160, I161, I162, I163, I64 |
| Alcoholism-related disorders | 980, 291.09-291.99, 303.09-303.99, 57109-57111, 57710 | F10 (except F10.0), G31.2, G62.1, G72.1, I 42.6, K29.2, K86.0, Z72.1. T51, K70 |
| Essential (primary) hypertension | 400.0–404.9 | I10-I15 |
| Angina pectoris | 413 | I20 |
| Lower urinary tract obstruction | 599.9 | N13, R30, R33,R35, R39. |
| Anemia | 280 | D50 |
| Venous thromboembolism | 450.99, 451.00, 451.08, 451.09, 451.90, 451.92, 451.99, 452- 453 | I80.1-9, I81-82, I26 |

| **Supplementary Table 2. Risk of lung cancer after syncope, by comorbid conditions. Data are standardized incidence ratios (SIRs) with 95% confidence intervals (CI)** | | | | | | | | | | | |
| --- | --- | --- | --- | --- | --- | --- | --- | --- | --- | --- | --- |
|  | **0-6 months** | | | **> 6-12 months** | | | **> 12 months** | | | **0-20 years** | |
| **Comorbidity** | **Observed** | **SIR (95% CI)** | **Observed** | | **SIR (95 % CI)** | **Observed** | | **SIR (95% CI)** | **Observed** | | **SIR (95% CI)** |
| **Total** | 366 | 2.7 (2.4-3.0) | 134 | | 1.1 (0.9-1.3) | 1655 | | 1.1 (1.0-1.1) | 2155 | | 1.2 (1.2-1.3) |
| **Head trauma** |  |  |  | |  |  | |  |  | |  |
| No | 288 | 2.8 (2.5-3.1) | 98 | | 1.0 (0.8-1.2) | 1261 | | 1.0 (1.0-1.1) | 1647 | | 1.1 (1.1-1.2) |
| Yes | 78 | 2.5 (2.0-3.2) | 36 | | 1.3 (0.9-1.8) | 394 | | 1.3 (1.2-1.5) | 508 | | 1.4 (1.3-1.6) |
| **Diabetes mellitus** |  |  |  | |  |  | |  |  | |  |
| No | 338 | 2.8 (2.5-3.1) | 125 | | 1.1 (0.9-1.3) | 1569 | | 1.1 (1.0-1.1) | 2032 | | 1.2 (1.2-1.3) |
| Yes | 28 | 2.3 (1.5-3.3) | 9 | | 0.8 (0.4-1.6) | 86 | | 1.0 (0.8-1.2) | 123 | | 1.1 (0.9-1.3) |
| **Myocardial infarction** |  |  |  | |  |  | |  |  | |  |
| No | 328 | 2.8 (2.5-3.1) | 122 | | 1.1 (0.9-1.3) | 1462 | | 1.0 (1.0-1.1) | 1912 | | 1.2 (1.1-1.2) |
| Yes | 38 | 2.3 (1.6-3.2) | 12 | | 0.8 (0.4-1.4) | 193 | | 1.3 (1.2-1.5) | 243 | | 1.4 (1.2-1.6) |
| **Heart failure** |  |  |  | |  |  | |  |  | |  |
| No | 335 | 2.8 (2.5-3.1) | 124 | | 1.1 (0.9-1.3) | 1540 | | 1.1 (1.0-1.1) | 1999 | | 1.2 (1.1-1.2) |
| Yes | 31 | 2.3 (1.6-3.2) | 10 | | 0.9 (0.4-1.6) | 115 | | 1.4 (1.2-1.7) | 156 | | 1.5 (1.2-1.7) |
| **Atrial fibrillation** |  |  |  | |  |  | |  |  | |  |
| No | 334 | 2.9 (2.6-3.2) | 107 | | 1.0 (0.8-1.2) | 1500 | | 1.1 (1.0-1.1) | 1941 | | 1.2 (1.2-1.3) |
| Yes | 32 | 1.6 (1.1-2.2) | 27 | | 1.5 (1.0-2.2) | 155 | | 1.1 (0.9-1.3) | 214 | | 1.2 (1.0-1.3) |
| **Valvular heart disease** |  |  |  | |  |  | |  |  | |  |
| No | 354 | 2.7 (2.5-3.0) | 129 | | 1.1 (0.9-1.3) | 1610 | | 1.1 (1.0-1.1) | 2093 | | 1.2 (1.1-1.3) |
| Yes | 12 | 2.1 (1.1-3.6) | 5 | | 1.0 (0.3-2.3) | 45 | | 1.2 (0.9-1.6) | 62 | | 1.3 (1.0-1.7) |
| **Chronic lower respiratory diseases** |  |  |  | |  |  | |  |  | |  |
| No | 303 | 2.5 (2.2-2.8) | 103 | | 0.9 (0.7-1.1) | 1448 | | 1.0 (1.0-1.1) | 1854 | | 1.1 (1.1-1.2) |
| Yes | 63 | 4.6 (3.6-5.9) | 31 | | 2.6 (1.8-3.7) | 207 | | 2.1 (1.8-2.4) | 301 | | 2.4 (2.2-2.7) |
| **Chronic kidney disease** |  |  |  | |  |  | |  |  | |  |
| No | 353 | 2.7 (2.4-3.0) | 128 | | 1.0 (0.9-1.3) | 1627 | | 1.1 (1.0-1.1) | 2108 | | 1.2 (1.1-1.2) |
| Yes | 13 | 3.1 (1.6-5.3) | 6 | | 1.7 (0.6-3.7) | 28 | | 1.2 (0.8-1.8) | 47 | | 1.5 (1.1-2.0) |
| **Obesity** |  |  |  | |  |  | |  |  | |  |
| No | 358-361 | 2.8 (2.5-3.1) | 130-133 | | 1.1 (0.9-1.3) | 1624 | | 1.1 (1.0-1.1) | 2115 | | 1.2 (1.2-1.3) |
| Yes | 5-8 | 1.3 (0.5-2.9) | 1-4 | | 0.7 (0.2-2.2) | 31 | | 0.8 (0.6-1.2) | 40 | | 0.9 (0.6-1.2) |
| **Alcoholism-related disorders** |  |  |  | |  |  | |  |  | |  |
| No | 340 | 2.6 (2.4-2.9) | 125 | | 1.0 (0.9-1.2) | 1499 | | 1.0 (1.0-1.1) | 1964 | | 1.1 (1.1-1.2) |
| Yes | 26 | 4.2 (2.8-6.2) | 9 | | 1.6 (0.7-3.1) | 156 | | 2.6 (2.2-3.0) | 191 | | 2.6 (2.3-3.0) |
| **Epilepsy** |  |  |  | |  |  | |  |  | |  |
| No | 355-358 | 2.7 (2.5-3.1) | 130-133 | | 1.1 (0.9-1.3) | 1590 | | 1.1 (1.0-1.1) | 2078 | | 1.2 (1.1-1.3) |
| Yes | 8-11 | 1.8 (0.8-3.4) | 1-4 | | 0.6 (0.1-1.9) | 65 | | 1.2 (0.9-1.6) | 77 | | 1.2 (1.0-1.5) |
| **Stroke** |  |  |  | |  |  | |  |  | |  |
| No | 330 | 2.8 (2.5-3.1) | 114 | | 1.0 (0.9-1.3) | 1487 | | 1.1 (1.0-1.1) | 1931 | | 1.2 (1.1-1.2) |
| Yes | 36 | 2.0 (1.4-2.8) | 20 | | 1.3 (0.8-1.9) | 168 | | 1.2 (1.0-1.4) | 224 | | 1.3 (1.1-1.5) |
| **Angina pectoris** |  |  |  | |  |  | |  |  | |  |
| No | 309 | 2.7 (2.4-3.1) | 106 | | 1.0 (0.8-1.2) | 1413 | | 1.1 (1.0-1.1) | 1828 | | 1.2 (1.1-1.2) |
| Yes | 57 | 2.6 (2.0-3.4) | 28 | | 1.4 (0.9-2.0) | 242 | | 1.2 (1.1-1.4) | 327 | | 1.3 (1.2-1.5) |
| **Hypertension** |  |  |  | |  |  | |  |  | |  |
| No | 289 | 2.8 (2.5-3.1) | 113 | | 1.1 (1.0-1.4) | 1450 | | 1.1 (1.1-1.2) | 1852 | | 1.2 (1.2-1.3) |
| Yes | 77 | 2.5 (2.0-3.2) | 21 | | 0.8 (0.5-1.2) | 205 | | 0.9 (0.8-1.0) | 303 | | 1.1 (1.0-1.2) |
| **Anemia** |  |  |  | |  |  | |  |  | |  |
| No | 359-362 | 2.7 (2.4-3.0) | 130-133 | | 1.1 (0.9-1.3) | 1642 | | 1.1 (1.0-1.1) | 2134 | | 1.2 (1.1-1.3) |
| Yes | 4-7 | 3.8 (1.5-7.9) | 1-4 | | 0.6 (0.0-3.6) | 13 | | 1.1 (0.6-1.8) | 21 | | 1.4 (0.8-2.1) |
| **Lower urinary tract obstruction** |  |  |  | |  |  | |  |  | |  |
| No | 346-349 | 2.7 (2.4-3.0) | 130-133 | | 1.1 (0.9-1.3) | 1612 | | 1.1 (1.0-1.1) | 2091 | | 1.2 (1.1-1.2) |
| Yes | 17-20 | 2.5 (1.5-4.0) | 1-4 | | 0.7 (0.2-1.8) | 43 | | 1.2 (0.9-1.6) | 64 | | 1.3 (1.0-1.7) |
| **Venous thromboembolism** |  |  |  | |  |  | |  |  | |  |
| No | 357 | 2.7 (2.5-3.1) | 127 | | 1.1 (0.9-1.3) | 1612 | | 1.1 (1.0-1.1) | 2096 | | 1.2 (1.2-1.3) |
| Yes | 9 | 1.8 (0.8-3.3) | 7 | | 1.5 (0.6-3.1) | 43 | | 1.1 (0.8-1.5) | 59 | | 1.2 (0.9-1.5) |

| **Supplementary Table 3. Risk of colorectal cancer after syncope, by comorbid conditions. Data are standardized incidence ratios (SIRs) with 95% confidence intervals (CI)** | | | | | | | | | | | |
| --- | --- | --- | --- | --- | --- | --- | --- | --- | --- | --- | --- |
|  | **0-6 months** | | | **> 6-12 months** | | | **> 12 months** | | | **0-20 years** | |
| **Comorbidity** | **Observed** | **SIR (95% CI)** | **Observed** | | **SIR (95 % CI)** | **Observed** | | **SIR (95% CI)** | **Observed** | | **SIR (95% CI)** |
| **Total** | 308 | 2.0 (1.8-2.2) | 149 | | 1.0 (0.9-1.2) | 1812 | | 1.0 (1.0-1.1) | 2269 | | 1.1 (1.1-1.2) |
| Head trauma |  |  |  | |  |  | |  |  | |  |
| No | 240 | 2.0 (1.8-2.3) | 110 | | 1.0 (0.8-1.2) | 1437 | | 1.0 (1.0-1.1) | 1787 | | 1.1 (1.0-1.1) |
| Yes | 68 | 1.9 (1.4-2.4) | 39 | | 1.2 (0.8-1.6) | 375 | | 1.1 (1.0-1.2) | 482 | | 1.2 (1.1-1.3) |
| Diabetes mellitus |  |  |  | |  |  | |  |  | |  |
| No | 275 | 1.9 (1.7-2.2) | 133 | | 1.0 (0.8-1.2) | 1695 | | 1.0 (1.0-1.1) | 2103 | | 1.1 (1.0-1.1) |
| Yes | 33 | 2.4 (1.7-3.4) | 16 | | 1.3 (0.8-2.2) | 117 | | 1.2 (1.0-1.4) | 166 | | 1.3 (1.1-1.5) |
| Myocardial infarction |  |  |  | |  |  | |  |  | |  |
| No | 265 | 1.9 (1.7-2.2) | 137 | | 1.1 (0.9-1.3) | 1652 | | 1.0 (1.0-1.1) | 2054 | | 1.1 (1.1-1.2) |
| Yes | 43 | 2.3 (1.7-3.2) | 12 | | 0.7 (0.4-1.3) | 160 | | 1.0 (0.8-1.1) | 215 | | 1.1 (0.9-1.2) |
| Heart failure |  |  |  | |  |  | |  |  | |  |
| No | 277 | 2.0 (1.8-2.2) | 139 | | 1.1 (0.9-1.3) | 1698 | | 1.0 (1.0-1.1) | 2114 | | 1.1 (1.1-1.1) |
| Yes | 31 | 1.9 (1.3-2.7) | 10 | | 0.7 (0.3-1.3) | 114 | | 1.1 (0.9-1.4) | 155 | | 1.2 (1.0-1.4) |
| Atrial fibrillation |  |  |  | |  |  | |  |  | |  |
| No | 261 | 2.0 (1.8-2.3) | 130 | | 1.1 (0.9-1.3) | 1665 | | 1.0 (1.0-1.1) | 2056 | | 1.1 (1.1-1.2) |
| Yes | 47 | 1.9 (1.4-2.6) | 19 | | 0.9 (0.5-1.4) | 147 | | 0.8 (0.7-1.0) | 213 | | 1.0 (0.8-1.1) |
| **Valvular heart disease** |  |  |  | |  |  | |  |  | |  |
| No | 295-298 | 2.0 (1.8-2.2) | 145-148 | | 1.1 (0.9-1.3) | 1757 | | 1.0 (1.0-1.1) | 2200 | | 1.1 (1.1-1.1) |
| Yes | 10-13 | 1.7 (0.9-3.0) | 1-4 | | 0.3 (0.0-1.2) | 55 | | 1.3 (0.9-1.6) | 69 | | 1.2 (0.9-1.5) |
| Chronic lower respiratory diseases |  |  |  | |  |  | |  |  | |  |
| No | 277 | 2.0 (1.8-2.2) | 135 | | 1.0 (0.9-1.2) | 1681 | | 1.0 (1.0-1.1) | 2093 | | 1.1 (1.0-1.1) |
| Yes | 31 | 2.1 (1.4-2.9) | 14 | | 1.1 (0.6-1.8) | 131 | | 1.2 (1.0-1.4) | 176 | | 1.3 (1.1-1.5) |
| Chronic kidney disease |  |  |  | |  |  | |  |  | |  |
| No | 295 | 2.0 (1.7-2.2) | 144 | | 1.0 (0.9-1.2) | 1791 | | 1.0 (1.0-1.1) | 2230 | | 1.1 (1.1-1.1) |
| Yes | 13 | 2.8 (1.5-4.8) | 5 | | 1.3 (0.4-3.0) | 21 | | 0.8 (0.5-1.3) | 39 | | 1.1 (0.8-1.6) |
| Obesity |  |  |  | |  |  | |  |  | |  |
| No | 296 | 2.0 (1.8-2.2) | 143 | | 1.0 (0.9-1.2) | 1759 | | 1.0 (1.0-1.1) | 2198 | | 1.1 (1.1-1.1) |
| Yes | 12 | 2.5 (1.3-4.4) | 6 | | 1.4 (0.5-3.1) | 53 | | 1.3 (1.0-1.8) | 71 | | 1.5 (1.2-1.9) |
| Alcoholism-related disorders |  |  |  | |  |  | |  |  | |  |
| No | 294 | 2.0 (1.8-2.2) | 144 | | 1.0 (0.9-1.2) | 1733 | | 1.0 (1.0-1.1) | 2171 | | 1.1 (1.0-1.1) |
| Yes | 14 | 2.4 (1.3-4.0) | 5 | | 0.9 (0.3-2.2) | 79 | | 1.3 (1.0-1.6) | 98 | | 1.4 (1.1-1.7) |
| Epilepsy |  |  |  | |  |  | |  |  | |  |
| No | 302-305 | 2.0 (1.8-2.3) | 145-148 | | 1.1 (0.9-1.3) | 1738 | | 1.0 (1.0-1.1) | 2188 | | 1.1 (1.1-1.1) |
| Yes | 3-6 | 1.1 (0.4-2.4) | 1-4 | | 0.2 (0.0-1.1) | 74 | | 1.3 (1.0-1.7) | 81 | | 1.2 (1.0-1.5) |
| Stroke |  |  |  | |  |  | |  |  | |  |
| No | 265 | 2.0 (1.7-2.2) | 127 | | 1.0 (0.8-1.2) | 1629 | | 1.0 (1.0-1.1) | 2021 | | 1.1 (1.0-1.1) |
| Yes | 43 | 2.0 (1.5-2.8) | 22 | | 1.2 (0.7-1.8) | 183 | | 1.1 (0.9-1.2) | 248 | | 1.2 (1.0-1.3) |
| Angina pectoris |  |  |  | |  |  | |  |  | |  |
| No | 257 | 2.0 (1.7-2.2) | 132 | | 1.1 (0.9-1.3) | 1599 | | 1.0 (1.0-1.1) | 1988 | | 1.1 (1.1-1.2) |
| Yes | 51 | 2.1 (1.5-2.7) | 17 | | 0.8 (0.4-1.2) | 213 | | 0.9 (0.8-1.1) | 281 | | 1.0 (0.9-1.1) |
| Hypertension |  |  |  | |  |  | |  |  | |  |
| No | 241 | 2.0 (1.8-2.3) | 114 | | 1.0 (0.8-1.2) | 1538 | | 1.0 (1.0-1.1) | 1893 | | 1.1 (1.0-1.1) |
| Yes | 67 | 1.9 (1.5-2.4) | 35 | | 1.1 (0.8-1.6) | 274 | | 1.0 (0.9-1.2) | 376 | | 1.1 (1.0-1.3) |
| Anemia |  |  |  | |  |  | |  |  | |  |
| No | 300 | 2.0 (1.8-2.2) | 144 | | 1.0 (0.9-1.2) | 1799 | | 1.0 (1.0-1.1) | 2243 | | 1.1 (1.1-1.1) |
| Yes | 8 | 3.4 (1.5-6.7) | 5 | | 2.5 (0.8-5.7) | 13 | | 0.8 (0.5-1.4) | 26 | | 1.3 (0.9-1.9) |
| Lower urinary tract obstruction |  |  |  | |  |  | |  |  | |  |
| No | 289 | 2.0 (1.7-2.2) | 144 | | 1.0 (0.9-1.2) | 1770 | | 1.0 (1.0-1.1) | 2203 | | 1.1 (1.1-1.1) |
| Yes | 19 | 2.5 (1.5-3.9) | 5 | | 0.8 (0.3-1.8) | 42 | | 1.0 (0.7-1.4) | 66 | | 1.2 (0.9-1.5) |
| **Venous thromboembolism** |  |  |  | |  |  | |  |  | |  |
| No | 294 | 2.0 (1.8-2.2) | 143 | | 1.0 (0.9-1.2) | 1756 | | 1.0 (1.0-1.1) | 2193 | | 1.1 (1.1-1.1) |
| Yes | 14 | 2.3 (1.3-3.9) | 6 | | 1.1 (0.4-2.4) | 56 | | 1.2 (0.9-1.5) | 76 | | 1.3 (1.0-1.6) |

| **Supplementary Table 4. Risk of breast cancer after syncope, by comorbid conditions. Data are standardized incidence ratios (SIRs) with 95% confidence intervals (CI)** | | | | | | | | | | |
| --- | --- | --- | --- | --- | --- | --- | --- | --- | --- | --- |
|  | **0-6 months** | | **> 6-12 months** | | | **> 12 months** | | | **0-20 years** | |
| **Comorbidity** | **Observed** | **SIR (95% CI)** | **Observed** | **SIR (95 % CI)** | **Observed** | | **SIR (95% CI)** | **Observed** | | **SIR (95% CI)** |
| **Total** | 140 | 1.2 (1.0-1.5) | 110 | 1.0 (0.8-1.2) | 1407 | | 1.0 (0.9-1.0) | 1657 | | 1.0 (1.0-1.1) |
| **Head trauma** |  |  |  |  |  | |  |  | |  |
| No | 107 | 1.2 (1.0-1.5) | 87 | 1.0 (0.8-1.3) | 1154 | | 1.0 (0.9-1.1) | 1348 | | 1.0 (1.0-1.1) |
| Yes | 33 | 1.3 (0.9-1.8) | 23 | 1.0 (0.6-1.5) | 253 | | 1.0 (0.9-1.1) | 309 | | 1.0 (0.9-1.1) |
| **Diabetes mellitus** |  |  |  |  |  | |  |  | |  |
| No | 125 | 1.2 (1.0-1.4) | 101 | 1.0 (0.8-1.2) | 1349 | | 1.0 (0.9-1.0) | 1575 | | 1.0 (1.0-1.1) |
| Yes | 15 | 1.9 (1.1-3.2) | 9 | 1.3 (0.6-2.4) | 58 | | 1.0 (0.7-1.2) | 82 | | 1.1 (0.9-1.4) |
| **Myocardial infarction** |  |  |  |  |  | |  |  | |  |
| No | 131 | 1.2 (1.0-1.5) | 101 | 1.0 (0.8-1.2) | 1339 | | 1.0 (0.9-1.0) | 1571 | | 1.0 (1.0-1.1) |
| Yes | 9 | 1.2 (0.6-2.4) | 9 | 1.4 (0.6-2.6) | 68 | | 1.1 (0.8-1.4) | 86 | | 1.1 (0.9-1.4) |
| **Heart failure** |  |  |  |  |  | |  |  | |  |
| No | 129 | 1.2 (1.0-1.4) | 101 | 1.0 (0.8-1.2) | 1350 | | 1.0 (0.9-1.0) | 1580 | | 1.0 (1.0-1.1) |
| Yes | 11 | 1.5 (0.7-2.7) | 9 | 1.4 (0.6-2.7) | 57 | | 1.2 (0.9-1.6) | 77 | | 1.3 (1.0-1.6) |
| **Atrial fibrillation** |  |  |  |  |  | |  |  | |  |
| No | 121 | 1.2 (1.0-1.4) | 94 | 1.0 (0.8-1.2) | 1323 | | 1.0 (0.9-1.1) | 1538 | | 1.0 (1.0-1.1) |
| Yes | 19 | 1.5 (0.9-2.3) | 16 | 1.4 (0.8-2.2) | 84 | | 0.9 (0.7-1.1) | 119 | | 1.0 (0.8-1.2) |
| **Valvular heart disease** |  |  |  |  |  | |  |  | |  |
| No | 136-139 | 1.2 (1.0-1.5) | 106-109 | 1.0 (0.8-1.2) | 1378 | | 1.0 (0.9-1.0) | 1621 | | 1.0 (1.0-1.1) |
| Yes | 1-4 | 0.8 (0.2-2.3) | 1-4 | 1.2 (0.3-3.1) | 29 | | 1.1 (0.8-1.7) | 36 | | 1.1 (0.8-1.5) |
| **Chronic lower respiratory diseases** |  |  |  |  |  | |  |  | |  |
| No | 130 | 1.2 (1.0-1.5) | 100 | 1.0 (0.8-1.2) | 1333 | | 1.0 (0.9-1.0) | 1563 | | 1.0 (1.0-1.1) |
| Yes | 10 | 1.1 (0.5-1.9) | 10 | 1.2 (0.6-2.1) | 74 | | 0.9 (0.7-1.2) | 94 | | 1.0 (0.8-1.2) |
| **Chronic kidney disease** |  |  |  |  |  | |  |  | |  |
| No | 136-139 | 1.2 (1.0-1.5) | 106-109 | 1.0 (0.8-1.2) | 1389 | | 1.0 (0.9-1.0) | 1633 | | 1.0 (1.0-1.1) |
| Yes | 1-4 | 0.9 (0.1-3.1) | 1-4 | 2.0 (0.5-5.1) | 18 | | 1.1 (0.7-1.8) | 24 | | 1.2 (0.8-1.8) |
| **Obesity** |  |  |  |  |  | |  |  | |  |
| No | 129-132 | 1.2 (1.0-1.4) | 106-109 | 1.0 (0.9-1.3) | 1367 | | 1.0 (0.9-1.0) | 1605 | | 1.0 (1.0-1.1) |
| Yes | 8-11 | 2.0 (0.9-3.8) | 1-4 | 0.7 (0.2-2.2) | 40 | | 1.0 (0.7-1.4) | 52 | | 1.1 (0.8-1.4) |
| **Alcoholism-related disorders** |  |  |  |  |  | |  |  | |  |
| No | 136-139 | 1.2 (1.0-1.5) | 106-109 | 1.0 (0.9-1.3) | 1370 | | 1.0 (0.9-1.0) | 1615 | | 1.0 (1.0-1.1) |
| Yes | 1-4 | 0.9 (0.2-2.5) | 1-4 | 0.6 (0.1-2.2) | 37 | | 1.0 (0.7-1.4) | 42 | | 0.9 (0.7-1.3) |
| **Epilepsy** |  |  |  |  |  | |  |  | |  |
| No | 136-139 | 1.2 (1.1-1.5) | 103-106 | 1.0 (0.8-1.2) | 1344 | | 1.0 (0.9-1.0) | 1585 | | 1.0 (1.0-1.1) |
| Yes | 1-4 | 0.8 (0.2-2.0) | 4-7 | 1.1 (0.3-2.5) | 63 | | 1.1 (0.8-1.4) | 72 | | 1.1 (0.8-1.3) |
| **Stroke** |  |  |  |  |  | |  |  | |  |
| No | 122 | 1.2 (1.0-1.4) | 97 | 1.0 (0.8-1.2) | 1305 | | 1.0 (0.9-1.0) | 1524 | | 1.0 (1.0-1.1) |
| Yes | 18 | 1.6 (0.9-2.5) | 13 | 1.2 (0.7-2.1) | 102 | | 1.1 (0.9-1.3) | 133 | | 1.1 (0.9-1.3) |
| **Angina pectoris** |  |  |  |  |  | |  |  | |  |
| No | 122 | 1.2 (1.0-1.4) | 94 | 1.0 (0.8-1.2) | 1252 | | 1.0 (0.9-1.0) | 1468 | | 1.0 (0.9-1.0) |
| Yes | 18 | 1.4 (0.8-2.3) | 16 | 1.4 (0.8-2.2) | 155 | | 1.3 (1.1-1.5) | 189 | | 1.3 (1.1-1.5) |
| **Hypertension** |  |  |  |  |  | |  |  | |  |
| No | 110 | 1.2 (1.0-1.5) | 84 | 1.0 (0.8-1.2) | 1227 | | 1.0 (0.9-1.0) | 1421 | | 1.0 (1.0-1.1) |
| Yes | 30 | 1.3 (0.9-1.9) | 26 | 1.2 (0.8-1.8) | 180 | | 1.0 (0.9-1.2) | 236 | | 1.1 (0.9-1.2) |
| **Anemia** |  |  |  |  |  | |  |  | |  |
| No | 136-139 | 1.2 (1.0-1.5) | 106-109 | 1.0 (0.8-1.2) | 1391-1397 | | 1.0 (0.9-1.0) | 1639 | | 1.0 (1.0-1.1) |
| Yes | 1-4 | 0.5 (0.0-2.9) | 1-4 | 0.6 (0.0-3.3) | 10-16 | | 1.0 (0.6-1.7) | 18 | | 0.9 (0.6-1.5) |
| **Lower urinary tract obstruction** |  |  |  |  |  | |  |  | |  |
| No | 136-139 | 1.2 (1.0-1.5) | 106-109 | 1.0 (0.9-1.2) | 1397-1403 | | 1.0 (0.9-1.0) | 1645 | | 1.0 (1.0-1.1) |
| Yes | 1-4 | 0.5 (0.0-2.8) | 1-4 | 0.6 (0.0-3.2) | 4-10 | | 0.7 (0.4-1.4) | 12 | | 0.7 (0.4-1.2) |
| **Venous thromboembolism** |  |  |  |  |  | |  |  | |  |
| No | 133-136 | 1.2 (1.0-1.5) | 136-139 | 1.0 (0.8-1.2) | 1370 | | 1.0 (0.9-1.0) | 1612 | | 1.0 (1.0-1.1) |
| Yes | 4-7 | 1.3 (0.4-3.1) | 1-4 | 0.9 (0.2-2.6) | 37 | | 1.2 (0.8-1.6) | 45 | | 1.1 (0.8-1.5) |

| **Supplementary Table 5. Risk of prostate cancer after syncope, by comorbid conditions. Data are standardized incidence ratios (SIRs) with 95% confidence intervals (CI)** | | | | | | | | | | |
| --- | --- | --- | --- | --- | --- | --- | --- | --- | --- | --- |
|  | **0-6 months** | | **> 6-12 months** | | | **> 12 months** | | | **0-20 years** | |
| **Comorbidity** | **Observed** | **SIR (95% CI)** | **Observed** | **SIR (95 % CI)** | **Observed** | | **SIR (95% CI)** | **Observed** | | **SIR (95% CI)** |
| **Total** | 228 | 1.7 (1.5-1.9) | 120 | 1.0 (0.8-1.1) | 1610 | | 1.0 (0.9-1.0) | 1958 | | 1.0 (1.0-1.1) |
| **Head trauma** |  |  |  |  |  | |  |  | |  |
| No | 174 | 1.7 (1.5-2.0) | 93 | 1.0 (0.8-1.2) | 1306 | | 1.0 (1.0-1.1) | 1573 | | 1.0 (1.0-1.1) |
| Yes | 54 | 1.6 (1.2-2.1) | 27 | 0.9 (0.6-1.3) | 304 | | 0.9 (0.8-1.0) | 385 | | 0.9 (0.9-1.0) |
| **Diabetes mellitus** |  |  |  |  |  | |  |  | |  |
| No | 210 | 1.7 (1.5-2.0) | 114 | 1.0 (0.8-1.2) | 1528 | | 1.0 (0.9-1.0) | 1852 | | 1.0 (1.0-1.1) |
| Yes | 18 | 1.2 (0.7-2.0) | 6 | 0.5 (0.2-1.0) | 82 | | 0.7 (0.6-0.9) | 106 | | 0.8 (0.6-0.9) |
| **Myocardial infarction** |  |  |  |  |  | |  |  | |  |
| No | 199 | 1.7 (1.5-2.0) | 108 | 1.0 (0.8-1.2) | 1439 | | 1.0 (1.0-1.1) | 1746 | | 1.0 (1.0-1.1) |
| Yes | 29 | 1.4 (0.9-2.0) | 12 | 0.6 (0.3-1.1) | 171 | | 0.8 (0.7-1.0) | 212 | | 0.9 (0.8-1.0) |
| **Heart failure** |  |  |  |  |  | |  |  | |  |
| No | 201 | 1.7 (1.5-2.0) | 108 | 1.0 (0.8-1.2) | 1528 | | 1.0 (0.9-1.0) | 1837 | | 1.0 (1.0-1.1) |
| Yes | 27 | 1.5 (1.0-2.3) | 12 | 0.8 (0.4-1.4) | 82 | | 0.7 (0.6-0.9) | 121 | | 0.8 (0.7-1.0) |
| **Atrial fibrillation** |  |  |  |  |  | |  |  | |  |
| No | 190 | 1.7 (1.5-2.0) | 92 | 0.9 (0.7-1.1) | 1454 | | 1.0 (0.9-1.0) | 1736 | | 1.0 (1.0-1.1) |
| Yes | 38 | 1.6 (1.1-2.2) | 28 | 1.3 (0.9-1.9) | 156 | | 0.9 (0.7-1.0) | 222 | | 1.0 (0.9-1.1) |
| **Valvular heart disease** |  |  |  |  |  | |  |  | |  |
| No | 219 | 1.7 (1.5-1.9) | 113 | 0.9 (0.8-1.1) | 1559 | | 1.0 (0.9-1.0) | 1891 | | 1.0 (1.0-1.1) |
| Yes | 9 | 1.3 (0.6-2.5) | 7 | 1.2 (0.5-2.5) | 51 | | 1.2 (0.9-1.5) | 67 | | 1.2 (0.9-1.5) |
| **Chronic respiratory diseases** |  |  |  |  |  | |  |  | |  |
| No | 198 | 1.6 (1.4-1.9) | 107 | 0.9 (0.8-1.2) | 1504 | | 1.0 (0.9-1.0) | 1809 | | 1.0 (1.0-1.1) |
| Yes | 30 | 2.0 (1.3-2.8) | 13 | 1.0 (0.5-1.7) | 106 | | 1.0 (0.8-1.2) | 149 | | 1.1 (0.9-1.3) |
| **Chronic kidney disease** |  |  |  |  |  | |  |  | |  |
| No | 219 | 1.7 (1.5-1.9) | 113 | 0.9 (0.8-1.1) | 1595 | | 1.0 (0.9-1.0) | 1927 | | 1.0 (1.0-1.1) |
| Yes | 9 | 1.7 (0.8-3.2) | 7 | 1.6 (0.6-3.2) | 15 | | 0.5 (0.3-0.9) | 31 | | 0.8 (0.5-1.1) |
| **Obesity** |  |  |  |  |  | |  |  | |  |
| No | 222-225 | 1.7 (1.5-1.9) | 116-119 | 1.0 (0.8-1.2) | 1589 | | 1.0 (0.9-1.0) | 1930 | | 1.0 (1.0-1.1) |
| Yes | 3-6 | 1.4 (0.5-3.1) | 1-4 | 0.3 (0.0-1.5) | 21 | | 0.6 (0.4-0.9) | 28 | | 0.7 (0.4-1.0) |
| **Alcoholism-related disorders** |  |  |  |  |  | |  |  | |  |
| No | 220-223 | 1.7 (1.5-2.0) | 116-119 | 1.0 (0.8-1.2) | 1549 | | 1.0 (0.9-1.0) | 1888 | | 1.0 (1.0-1.1) |
| Yes | 5-8 | 0.8 (0.3-1.8) | 1-4 | 0.4 (0.1-1.3) | 61 | | 0.7 (0.6-1.0) | 70 | | 0.7 (0.6-0.9) |
| **Epilepsy** |  |  |  |  |  | |  |  | |  |
| No | 221 | 1.7 (1.5-1.9) | 115 | 0.9 (0.8-1.1) | 1569 | | 1.0 (0.9-1.0) | 1905 | | 1.0 (1.0-1.1) |
| Yes | 7 | 1.5 (0.6-3.0) | 5 | 1.1 (0.4-2.7) | 41 | | 0.8 (0.6-1.1) | 53 | | 0.9 (0.7-1.1) |
| **Stroke** |  |  |  |  |  | |  |  | |  |
| No | 200 | 1.7 (1.5-2.0) | 105 | 1.0 (0.8-1.2) | 1467 | | 1.0 (0.9-1.1) | 1772 | | 1.0 (1.0-1.1) |
| Yes | 28 | 1.4 (0.9-2.0) | 15 | 0.8 (0.5-1.3) | 143 | | 0.8 (0.7-1.0) | 186 | | 0.9 (0.8-1.0) |
| **Angina pectoris** |  |  |  |  |  | |  |  | |  |
| No | 185 | 1.7 (1.5-2.0) | 100 | 1.0 (0.8-1.2) | 1385 | | 1.0 (1.0-1.1) | 1670 | | 1.0 (1.0-1.1) |
| Yes | 43 | 1.6 (1.2-2.2) | 20 | 0.8 (0.5-1.3) | 225 | | 0.9 (0.8-1.0) | 288 | | 0.9 (0.8-1.0) |
| **Hypertension** |  |  |  |  |  | |  |  | |  |
| No | 176 | 1.7 (1.5-2.0) | 96 | 1.0 (0.8-1.2) | 1379 | | 1.0 (0.9-1.0) | 1651 | | 1.0 (1.0-1.1) |
| Yes | 52 | 1.6 (1.2-2.1) | 24 | 0.8 (0.5-1.2) | 231 | | 0.9 (0.8-1.1) | 307 | | 1.0 (0.9-1.1) |
| **Anemia** |  |  |  |  |  | |  |  | |  |
| No | 224-227 | 1.7 (1.5-1.9) | 120 | 1.0 (0.8-1.2) | 1592-1595 | | 1.0 (0.9-1.0) | 1939 | | 1.0 (1.0-1.1) |
| Yes | 1-4 | 2.4 (0.7-6.1) | 0 | 0.0 (.-.) | 15-18 | | 1.6 (0.9-2.6) | 19 | | 1.5 (0.9-2.4) |
| **Lower urinary tract obstruction** |  |  |  |  |  | |  |  | |  |
| No | 191 | 1.5 (1.3-1.8) | 106 | 0.9 (0.8-1.1) | 1555 | | 1.0 (0.9-1.0) | 1852 | | 1.0 (1.0-1.1) |
| Yes | 37 | 3.3 (2.3-4.6) | 14 | 1.5 (0.8-2.5) | 55 | | 0.9 (0.7-1.2) | 106 | | 1.3 (1.1-1.6) |
| **Venous thromboembolism** |  |  |  |  |  | |  |  | |  |
| No | 213-216 | 1.6 (1.4-1.9) | 116-119 | 1.0 (0.8-1.2) | 1570 | | 1.0 (0.9-1.0) | 1902 | | 1.0 (1.0-1.1) |
| Yes | 12-15 | 2.4 (1.3-4.1) | 1-4 | 0.4 (0.1-1.4) | 40 | | 0.9 (0.6-1.2) | 56 | | 1.0 (0.8-1.3) |
